# Pre-hospital point-of-care troponin measurement: a clinical example of its additional value

**DOI:** 10.1007/s12471-020-01434-w

**Published:** 2020-06-08

**Authors:** G. W. A. Aarts, K. van der Wulp, C. Camaro

**Affiliations:** Department of Cardiology, Radboud University Medical Centre, Nijmegen, The Netherlands

**Keywords:** HEART score, Point-of-care troponin, Non-ST-segment elevation, Acute coronary syndrome, Randomised trial

## Abstract

In the majority of patients with chest pain, an acute coronary syndrome (ACS) can be ruled out. However, early recognition of an ACS is required in order to start treatment as soon as possible and reduce risks associated with myocardial ischaemia. Because of the lack of pre-hospital protocols to rule out an ACS, patients with a suspected ACS are transported to the emergency department, where the HEART score can be used to estimate the risk of major adverse cardiac events (MACE). Patients with a low HEART score have a low risk of MACE. A point-of-care (POC) troponin measurement enables ambulance paramedics to calculate the HEART score in the pre-hospital setting. POC troponin measurement and HEART score assessment have several potential advantages, including early recognition of an ACS and identification of high-risk patients before hospital arrival. Moreover, pre-hospital rule-out of an ACS could prevent unnecessary emergency department visits. The safety and cost-effectiveness of referring low-risk patients with a normal POC troponin value to the general practitioner are currently being investigated in the ARTICA randomised trial. This point-of-view article demonstrates one of the potential advantages of early detection of an ACS.

A 49-year-old man called the emergency services because of chest pain that had been present for 10 h. Based on the patient’s symptoms, age, cardiovascular risk profile and electrocardiogram (ECG), he was classified as low risk by the ambulance paramedics. On-site, a point-of-care (POC) troponin measurement was performed to rule out an acute coronary syndrome (ACS). What do we currently know about the pre-hospital assessment of POC troponin and what does it add to current daily practice? The aim of this point-of-view article is to give an overview of the current knowledge of pre-hospital POC troponin measurement and to illustrate one of its potential advantages.

## The clinical problem

In patients with chest pain, one of the main diagnostic concerns is whether the pain is due to an ACS. Early recognition of an ACS is essential, since patients with ongoing ischaemia are at risk of life-threatening arrhythmias [1]. Fifteen percent of patients with acute onset of chest pain are known to have an ACS, and pharmacological therapy should be started as soon as possible when an ACS is diagnosed [2, 3]. It is noteworthy that in the majority (85%) of patients with chest pain, an ACS can be ruled out. Yet, in order to rule out an ACS, a combination of electrocardiography, clinical evaluation and cardiac troponin measurements is required [1]. Because of the lack of pre-hospital protocols to rule out an ACS, high numbers of patients are transported to the emergency department (ED) when an ACS is suspected, even when the a priori risk for an ACS is low.

To estimate the risk for major adverse cardiac events (MACE) in chest pain patients presenting to the ED, the HEART (history, electrocardiogram, age, risk factors and troponin) score can be used. This score was designed to identify patients eligible for early discharge from the ED [4]. Low-risk patients, i.e. patients with a HEART score of ≤3, comprise 30% of all chest pain patients and have a 1.9% risk of developing short-term (30 days to 6 weeks) MACE [5, 6]. The estimated risk of MACE decreases to 0.8% if the troponin level is below the 99th percentile [6]. The use of a POC troponin measurement enables ambulance paramedics to calculate an on-site HEART score and reconsider immediate transportation to the ED [7]. The POC troponin T assay that has been investigated in this setting (Roche cobas h232) has a detection width of 40–2,000 ng/l (lower results are reported as a value <40 ng/l). The within-series coefficient of variation is 9.3% in the low concentration range (40–200 ng/l) and yields very good analytical concordance with high-sensitivity troponin T [8]. The final result is available after a maximum of 12 min.

**Fig. 1:**
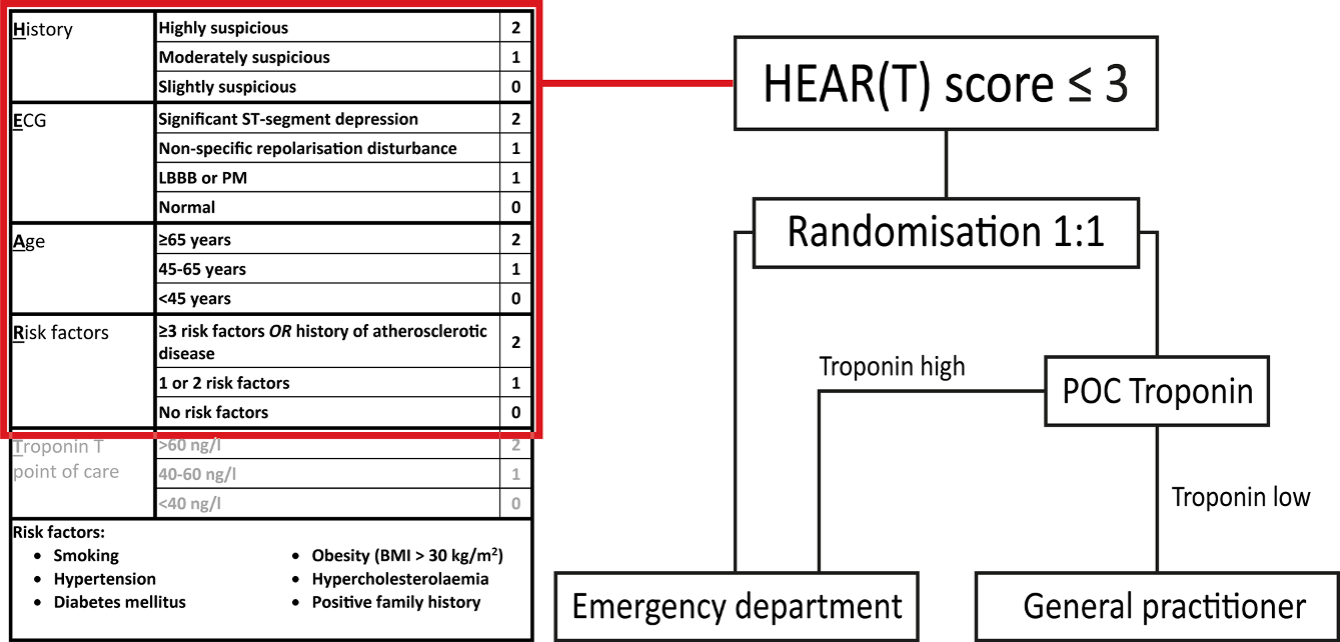
Design of the ARTICA trial. (*ECG* electrocardiogram, *LBBB* left bundle branch block, *PM* pacemaker, *BMI* body mass index, *POC* point-of-care)

Pre-hospital POC troponin measurement has several potential advantages. First, it might lead to early recognition of an ACS and could be used to identify high-risk patients even before hospital arrival [9]. Second, ruling out an ACS at home with the HEART score and a POC troponin measurement could prevent unnecessary ED visits and subsequently reduce healthcare consumption and costs. The safety and cost-effectiveness of referring low-risk patients with a normal POC troponin T value (<40 ng/l) to the general practitioner instead of the ED are currently being investigated in the ARTICA randomised trial (Fig. 1; [10]).

## Case description

**Fig. 2:**
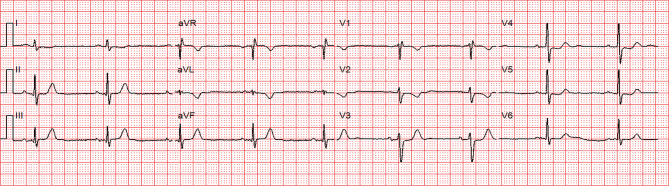
Electrocardiogram in the ambulance

**Fig. 3:**
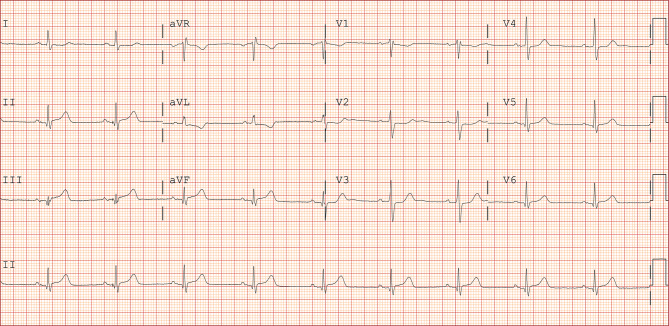
Electrocardiogram in the emergency department, 15 min after arrival

**Fig. 4:**
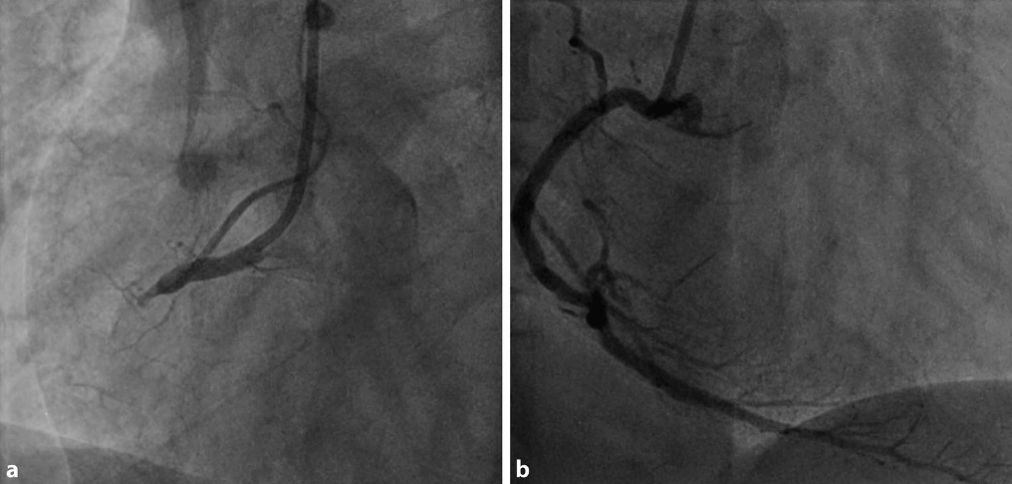
Coronary angiography. **a** Proximal occlusion of the right coronary artery. **b** After percutaneous coronary intervention

The ambulance paramedics evaluated the aforementioned patient and assessed the HEAR score (HEART score without the troponin component); history was interpreted as moderately suspicious (1 point), the ECG (Fig. 2) was interpreted as normal (0 points), age was 49 years (1 point) and the patient’s only risk factor was smoking (1 point). The patient gave signed informed consent to participate in the ARTICA trial and was randomised to the intervention arm (completion of the HEART score at home with a POC troponin T measurement, instead of immediate transport to the ED for further evaluation). The on-site POC troponin T value proved to be elevated (214 ng/l) and, following study protocol, the patient was immediately transported to the ED. At the ED, the first ECG was similar to the pre-hospital ECG. Yet, awaiting clinical evaluation at the ED, 15 min later the ECG started to show subtle ST-segment elevations (Fig. 3). Emergency coronary angiography (CAG) (<30 min after ED admission) revealed a proximal occlusion of the right coronary artery, for which percutaneous coronary intervention (PCI) was performed successfully (Fig. 4). According to the current European Society of Cardiology guidelines for the management of acute myocardial infarction in patients presenting with ST-segment elevation, PCI is the preferred reperfusion strategy in patients with an ST-segment elevation myocardial infarction (STEMI), provided it can be performed within 120 min of the STEMI diagnosis [11]. Moreover, shortening of delays from diagnosis to treatment in STEMI patients is associated with lower mortality rates [12]. In this patient, the time from STEMI diagnosis to CAG was 15 min and would have been much longer if the patient had been transported to a non-PCI centre first. Hypothetically, pre-hospital identification of patients who should be transported to a primary PCI centre immediately could reduce diagnosis-to-treatment time and subsequently reduce mortality. Therefore, future research should aim at investigating whether patients with elevated pre-hospital POC troponin levels benefit from immediate transport to a centre with PCI facilities.

## Conclusion

This case illustrates that pre-hospital POC troponin measurement can result in early detection of myocardial infarction and reduce diagnosis-to-treatment time, even in low-risk patients with initially non-suspicious ECGs. Further research is needed to investigate whether chest pain patients with elevated pre-hospital POC troponin levels benefit from immediate transport to a centre with PCI facilities. In the meantime, the results of the ARTICA randomised trial (Netherlands Trial Register NL7148) have to be awaited to establish whether POC troponin measurement for early rule-out of an ACS in low-risk patients is cost-effective and safe.

## References

[1] Roffi M, Patrono C, Collet JP, Mueller C, Valgimigli M, Andreotti F (2015). 2015 ESC guidelines for the management of acute coronary syndromes in patients presenting without persistent ST-segment elevation. Rev Esp Cardiol (Engl Ed).

[2] Mol KA, Rahel BM, Meeder JG, van Casteren BC, Doevendans PA, Cramer MJ (2016). Delays in the treatment of patients with acute coronary syndrome: focus on pre-hospital delays and non-ST-elevated myocardial infarction. Int J Cardiol.

[3] Nasrallah N, Steiner H, Hasin Y (2011). The challenge of chest pain in the emergency room: now and the future. Eur Heart J.

[4] Six AJ, Backus BE, Kelder JC (2008). Chest pain in the emergency room: value of the HEART score. Neth Heart J.

[5] Van Den Berg P, Body R (2018). The HEART score for early rule out of acute coronary syndromes in the emergency department: a systematic review and meta-analysis. Eur Heart J Acute Cardiovasc Care.

[6] Laureano-Phillips J, Robinson RD, Aryal S, Blair S, Wilson D, Boyd K (2019). HEART score risk stratification of low-risk chest pain patients in the emergency department: a systematic review and meta-analysis. Ann Emerg Med.

[7] van Dongen DN, Tolsma RT, Fokkert MJ, Badings EA, van der Sluis A, Slingerland RJ (2018). Pre-hospital risk assessment in suspected non-ST-elevation acute coronary syndrome: a prospective observational study. Eur Heart J Acute Cardiovasc Care.

[8] Jungbauer C, Hupf J, Giannitsis E, Frick J, Slagman A, Ehret C (2017). Analytical and clinical validation of a point-of-care cardiac troponin T test with an improved detection limit. Clin Lab.

[9] Rasmussen MB, Stengaard C, Sorensen JT, Riddervold IS, Hansen TM, Giebner M (2017). Predictive value of routine point-of-care cardiac troponin T measurement for prehospital diagnosis and risk-stratification in patients with suspected acute myocardial infarction. Eur Heart J Acute Cardiovasc Care.

[10] Aarts GWA, Camaro C, van Geuns RJ, Cramer E, van Kimmenade RRJ, Damman P (2020). Acute rule-out of non-ST-segment elevation acute coronary syndrome in the (pre)hospital setting by HEART score assessment and a single point-of-care troponin: rationale and design of the ARTICA randomised trial. BMJ Open.

[11] Ibanez B, James S, Agewall S, Antunes MJ, Bucciarelli-Ducci C, Bueno H (2017). 2017 ESC Guidelines for the management of acute myocardial infarction in patients presenting with ST-segment elevation. Rev Esp Cardiol (Engl Ed).

[12] Nallamothu BK, Normand SL, Wang Y, Hofer TP, Brush JE, Messenger JC (2015). Relation between door-to-balloon times and mortality after primary percutaneous coronary intervention over time: a retrospective study. Lancet.

